# Characterization and downstream mannose phosphorylation of human recombinant α-L-iduronidase produced in Arabidopsis *complex glycan-deficient* (*cgl*) seeds

**DOI:** 10.1111/pbi.12096

**Published:** 2013-07-31

**Authors:** Xu He, Owen Pierce, Thomas Haselhorst, Mark von Itzstein, Daniel Kolarich, Nicolle H Packer, Tracey M Gloster, David J Vocadlo, Yi Qian, Doug Brooks, Allison R Kermode

**Affiliations:** 1 Department of Biological Sciences, Simon Fraser UniversityBurnaby, BC, Canada; 2 Institute for Glycomics, Griffith UniversitySouthport, Qld, Australia; 3 Department of Chemistry and Biomolecular Sciences, Macquarie UniversitySydney, NSW, Australia; 4 Department of Chemistry, Simon Fraser UniversityBurnaby, BC, Canada; 5 Department of Internal Medicine, Washington University School of MedicineSt. Louis, MO, USA; 6 Sansom Institute, School of Pharmacy and Medical Sciences, University of South AustraliaAdelaide, SA, Australia

**Keywords:** Arabidopsis *cgl* mutant, mucopolysaccharidosis I, human α-L-iduronidase, mannose-6-phosphate recognition marker, *N*-glycosylation

## Abstract

Mucopolysaccharidosis (MPS) I is a lysosomal storage disease caused by a deficiency of α-L-iduronidase (IDUA) (EC 3.2.1.76); enzyme replacement therapy is the conventional treatment for this genetic disease. *Arabidopsis cgl* mutants are characterized by a deficiency of the activity of *N*-acetylglucosaminyl transferase I (EC 2.4.1.101), the first enzyme in the pathway of hybrid and complex *N*-glycan biosynthesis. To develop a seed-based platform for the production of recombinant IDUA for potential treatment of MPS I, *cgl* mutant seeds were generated to express human IDUA at high yields and to avoid maturation of the *N*-linked glycans on the recombinant human enzyme. Enzyme kinetic data showed that *cgl*-IDUA has similar enzymatic properties to the commercial recombinant IDUA derived from cultured Chinese hamster ovary (CHO) cells (Aldurazyme™). The *N*-glycan profile showed that *cgl*-derived IDUA contained predominantly high-mannose-type *N*-glycans (94.5%), and the residual complex/hybrid *N*-glycan-containing enzyme was efficiently removed by an additional affinity chromatography step. Furthermore, purified *cgl*-IDUA was amenable to sequential *in vitro* processing by soluble recombinant forms of the two enzymes that mediate the addition of the mannose-6-phosphate (M6P) tag in mammalian cells—UDP-GlcNAc:lysosomal enzyme *N*−acetylglucosamine (GlcNAc)−1−phosphotransferase—and GlcNAc−1−phosphodiester α−*N*−acetylglucosaminidase (the ‘uncovering enzyme’). *Arabidopsis* seeds provide an alternative system for producing recombinant lysosomal enzymes for enzyme replacement therapy; the purified enzymes can be subjected to downstream processing to create the M6P, a recognition marker essential for efficient receptor-mediated uptake into lysosomes of human cells.

## Introduction

Lysosomal storage diseases (LSDs) collectively represent over 70 inherited metabolic disorders. The underlying etiology of many LSDs involves a deficiency of a single acid hydrolase. Enzyme replacement therapy (ERT) is the conventional treatment. While some enzyme replacement therapeutics are in the ‘pipeline’ and are under different phases of clinical evaluation (van Gelder *et al*., 2012), to date, these therapeutics have been registered for only a few of the LSDs: Gaucher disease, Fabry disease, Pompe disease and mucopolysaccharidoses (MPS I, II, and VI). ERT is a process that takes advantage of plasma membrane receptor uptake to deliver recombinant enzyme into a patient's cells following intravenous infusion of the replacement enzyme (Neufeld, 2011). Mucopolysaccharidosis I (MPS I) disease is an LSD characterized by the deficiency of α-L-iduronidase (IDUA), an enzyme involved in the stepwise degradation of the glycosaminoglycans heparan sulphate and dermatan sulphate. Because of the widespread distribution of these glycosaminoglycans in tissues and organs, severely affected humans with no residual α-L-iduronidase typically die in early childhood due to profound skeletal, cardiac and neurological disturbances (Clarke, 2008). The current approved ERT for MPS I (*Aldurazyme*™ or *Laronidase*), based on the recombinant IDUA from Chinese hamster ovary (CHO) cells, is prohibitively expensive, costing approximately US$450 000 per year for an average 12-year-old child. As costs are calculated on the basis of the weight of an individual, treatments for teenagers and adults are even higher.

Plant-based systems offer an alternative protein production platform that could reduce the costs associated with these therapies. An enzyme therapeutic for Gaucher disease generated by a plant platform (carrot suspension cell cultures) was approved by the US Food and Drug Administration in 2012 (Maxmen, 2012; Shaaltiel *et al*., 2007) and represents the first plant-based recombinant therapeutic approved for parenteral administration. Some of the advantages of plant expression systems include the following: relatively low production costs due to an ability to rapidly accumulate biomass and a lack of susceptibility to contamination by human pathogens as can occur in mammalian expression systems, such as CHO cells or human fibroblasts. Seeds, as natural depots for stable accumulation of proteins, offer additional advantages as a ‘host’ platform: recombinant proteins are usually stable in the mature seeds placed in conventional (cool, dry) storage conditions, and the seeds remain viable for several years. Thus, seeds serve as a stable repository of the therapeutic protein providing the convenient option of temporally separating the production of the recombinant protein from its purification and downstream processing (Kermode, 2012).

Nonetheless, the use of plants as platforms for the production of recombinant lysosomal enzymes suitable for ERT poses at least three technical challenges. First, sufficient yields of the recombinant enzyme must be obtained to render economic viability to the production platform. Second, a nonimmunogenic and active recombinant protein must be generated. While the initial steps of protein *N*-glycosylation and subsequent *N*-glycan trimming that are essential for proper protein folding are common to plant and animal cells, differences occur in *N*-glycan maturation as proteins transit through the distal compartments of the Golgi complex (*medial*-, *trans*- and *trans*-Golgi networks). In these compartments, enzymes convert the high-mannose-type *N*-glycans of proteins to complex *N*-glycans by a series of sequential reactions that rely on the accessibility of the glycan chain(s) to the Golgi processing machinery (Gomord *et al*., 2010; Kermode, 1996). In mammalian cells, core α1,6 fucose residues and terminal sialic acid residues are commonly added, whereas bisecting β1,2 xylose and core α1,3 fucose residues are assembled onto the trimmed *N*-glycans of plant-synthesized proteins. The presence of β1,2 xylose and/or α1,3 fucose residues on plant-produced pharmaceuticals can render the product immunogenic, which is a concern for any therapeutic destined for parenteral administration (reviewed in Gomord *et al*., 2010).

Third, there are challenges associated with generating a therapeutically efficacious product; the parenterally administered recombinant enzyme must have suitable targeting signals for endocytosis into patient cells and for intracellular delivery to the lysosome. For most lysosomal enzymes (e.g. α-L-iduronidase and several others), this requires the cellular recognition marker, mannose-6-phosphate (M6P), on the replacement protein.

In mammalian cells, Golgi-specific enzymatic processing mediates M6P elaboration, a process involving the modification of select terminal mannose residues of some of the *N*-glycans of lysosomal hydrolases. Thus, lysosomal hydrolases typically possess various *N*-glycans, namely those of the high-mannose-, complex- or M6P-terminated types. The M6P recognition motif normally mediates the targeting of soluble lysosomal enzymes from the *trans*-Golgi network to the lysosome, but can also mediate the internalization of inappropriately secreted lysosomal enzymes via the so-called ‘salvage’ pathway. ERT for LSDs largely exploits this endogenous ‘salvage’ (uptake) pathway of human cells, which is effected by cell surface endocytic receptors, such as the M6P receptors (reviewed in Gary-Bobo *et al*., 2007). ‘Retrieval’ or sequestration of an extracellular (e.g. recombinant) lysosomal enzyme due to recognition of its M6P marker can result in delivery to the lysosome by receptor-mediated endocytosis via early and late endosomes, enabling the correction of an enzyme deficiency. Thus, a key challenge lies in the fact that plant cells do not have the endogenous machinery to create the M6P tag on a recombinant protein; a viable option is to create this on the purified protein *in vitro*, that is, as part of downstream processing.

In this study, we address the three challenges associated with plant-based platforms for the production of recombinant lysosomal enzymes destined for ERT, focussing on human IDUA. Through selection and inbreeding, we identified an *Arabidopsis thaliana* (Arabidopsis) seed line that significantly exceeds our previously reported yields of recombinant human IDUA by at least threefold (Downing *et al*., 2006, 2007); in this line, IDUA represented 5.7% of the total soluble seed protein (TSP), and the yield was stable through T3 and T4 generations. This Arabidopsis seed line represents a mutant background (the *complex glycan-deficient* or *cgl1* C5 mutant), in which *N*-glycan maturation is avoided due to a deficiency of the Golgi enzyme *N*-acetylglucosamine transferase I (GnT I); importantly, we show by graphitized carbon liquid chromatography–tandem mass spectrometry that high-mannose-type *N*-glycans accounted for 94.5% of the *N*-glycans on the *cgl*-IDUA. Kinetic parameters of the *cgl*-derived IDUA were comparable with those of Aldurazyme™ (the commercial CHO cell–derived IDUA); we further demonstrate that the plant recombinant enzyme was amenable to *in vitro* downstream processing using two recombinant soluble human enzymes: GlcNAc-1-phosphotransferase and the ‘uncovering enzyme’, to generate the M6P recognition motif.

Our results show that Arabidopsis *cgl* seeds provide a viable production system for recombinant human IDUA, generating enzyme that is potentially suitable for treating patients with MPS I.

## Results

### Arabidopsis *cgl* seeds accumulate high levels of active α-L-iduronidase

**Figure 1:**
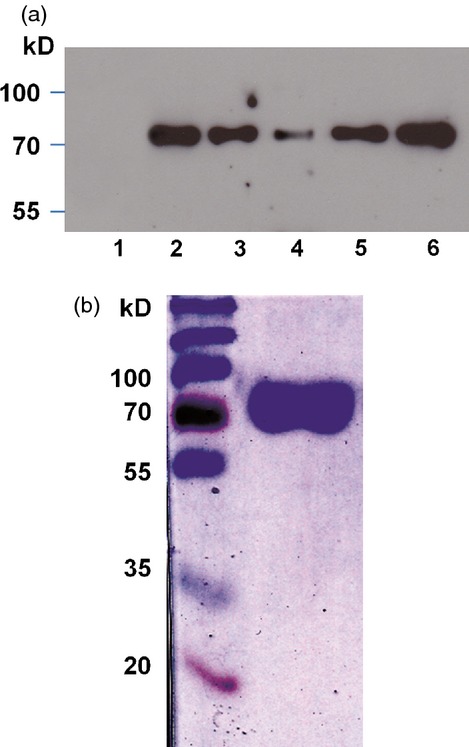
α-L-Iduronidase (IDUA) produced in Arabidopsis *cgl* seeds. (a) Western blot analysis of IDUA produced in T3 seeds of 3 high-expressing lines. Lanes 1–4: 0.4 μg of total soluble protein was loaded from the crude extracts of *cgl* untransformed seeds (lane 1) and from seeds of transformed lines A5.5, A4.7 and A6.3, respectively (lanes 2–4). Lanes 5–6 show 20 ng and 50 ng of purified *cgl*-IDUA (from line A4.7), respectively. (b) Five micrograms of affinity-purified *cgl*-IDUA (line A4.7) was loaded on a 10% gel, and after SDS-PAGE, the gel was stained with Coomassie Brilliant Blue and destained. Molecular weights of the prestained protein markers are indicated on the left.

Previously, we reported that using the promoter and other regulatory sequences of the *Phaseolus vulgaris arcelin 5-I* gene, three transgenic Arabidopsis *cgl* lines with exceptional activities were identified; these possessed IDUA activities of 820 ± 63, 745 ± 75 and 423 ± 19 units/mg TSP (Downing *et al*., 2007; lines A5.5, A4.7 and A6.3, respectively). The highest yielding line accumulated IDUA to approximately 18 μg IDUA/mg TSP (Downing *et al*., 2007). Here we further improved the yield by selfing of plants and by further selection. T2 seeds from the three lines were germinated on selection media (25 mg/L kanamycin in half-strength Murashige and Skoog [MS] medium). Ten to 15 transgenic seedlings from each line were grown to maturity and selfed to obtain T3 seeds. Protein was extracted from each seed stock, and the expression levels were determined by activity assays and Western blot analyses. Ten transgenic seedlings from each of the highest expressers were selfed to generate T4 seeds. Interestingly, the highest expressing line of T3 seeds accumulated IDUA at 11.2% TSP (Table 1 and Figure 1a, lane 2), but the expression level of the subsequent generation (T4) decreased significantly (Table 1). Although we have not determined the zygosity of the transgene, the highest yielding line (with relatively stable trans-generation expression) accumulated IDUA at 5.7% TSP (Table 1).

**Table 1:** Accumulation of IDUA (as% total soluble seed protein (TSP) or μg/mg seeds) in transgenic Arabidopsis seed lines

Line	T2		T3 (±SD)		T4 (±SD)	
	%TSP	μg/mg	%TSP	μg/mg	%TSP	μg/mg
A4.7	1.7	2.7	7.2 ± 0.6	9.8 ± 0.57	5.7 ± 0.4	8.0 ± 0.8
A5.5	1.8	3.0	11.2 ± 2.7	16.3 ± 2.1	2.5 ± 0.14	4.0 ± 0.55
A6.3	0.94	1.6	2.6 ± 0.23	4.3 ± 0.40	1.6 ± 0.08	2.3 ± 0.18

TSP, total soluble protein; SD, standard deviation of three replicates. For T3 seeds, only the highest line among the 15 siblings is presented; for T4 seeds, the accumulation levels were measured in a seed stock pool made up of seeds from 10–15 transgenic plants.

### *Cgl*-derived IDUA contains predominantly high-mannose-type *N*-glycans

There are six consensus sites for *N*-linked glycosylation on α-L-iduronidase (Asn-X-Ser/Thr); all sites are utilized in human cells (Kakkis, 2005). In CHO cells hosting production of the recombinant protein, the enzyme is secreted, and all six glycosylation sites are used, but the *N*-linked glycans themselves display high intrasite heterogeneity (Zhao *et al*., 1997). Some of the *N*-linked glycans of the mature enzyme remain in a high-mannose form (Asn 372; Asn 415 is mixed high mannose and complex); at least two of the sites are modified to complex forms (Asn 110 and Asn 190); two carry M6P tags for receptor-mediated uptake/lysosome delivery (Asn 336 and Asn 451) (Zhao *et al*., 1997).

**Figure 2:**
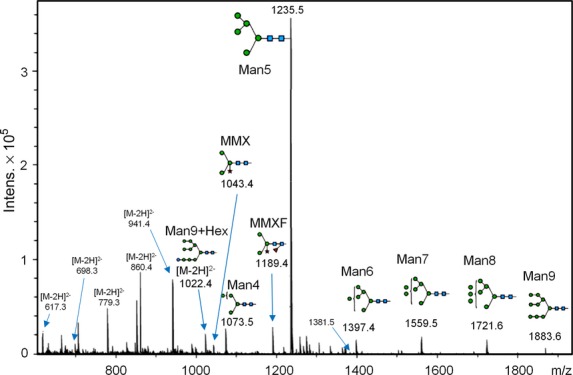
*N*-glycans of *cgl*-α-L-iduronidase (IDUA). Summed mass spectra of *N*-glycans from *cgl*-IDUA. Signals were identified as [M–H]^−^ signals unless stated otherwise. Oligomannosidic structures comprised more than 90% of the structures identified (see also Table 2).

**Figure 3:**
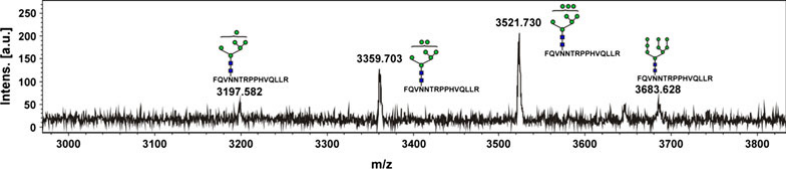
High-mannose type *N*-glycan at site Asn 372 on recombinant *cgl*-IDUA protein (glycopeptide 369–383).

In the Arabidopsis *cgl* mutant, the *N*-glycan status of the recombinant protein appears to depend on both the target protein and the plant tissues that ‘host’ its expression (see Discussion). As shown in Figure 2 and Table 2, 94.5% of the *N*-glycan structures detected in the purified *cgl*-IDUA (Figure 1b) by carbon LC MS/MS were of the oligomannosidic type containing 1–7 hexose residues in addition to the pentasaccharide *N*-glycan core. Among these structures, a Man5 structure (i.e. Man_5_GlcNAc_2_) was the most abundant (50.6%). Complex and hybrid structures containing core α 1,3 fucose and/or β 1,2 linked xylose accounted for 5.5% of the total structures detected (Table 2). One glycopeptide could be identified from the *cgl*-IDUA samples, indicating incomplete digestion with PNGase A. Nevertheless, this allowed the identification of high-mannose-type *N*-glycosylation at site Asn 372 (Figure 3).

**Table 2:** *N*-glycans identified in *cgl*-IDUA

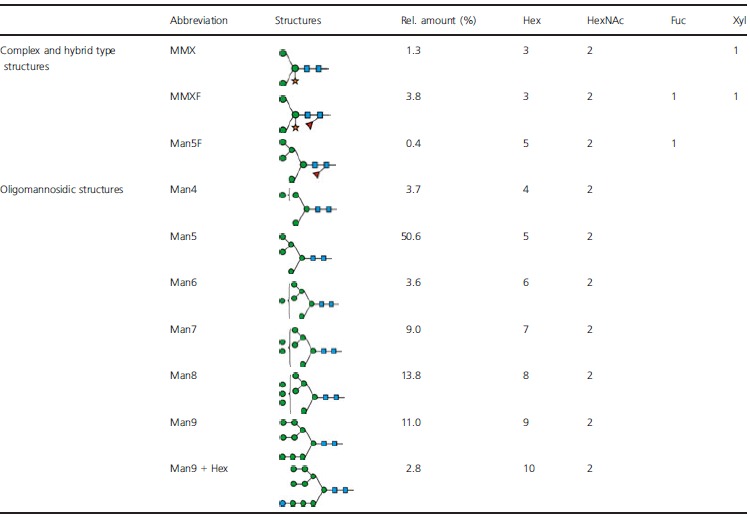

**Figure 4:**
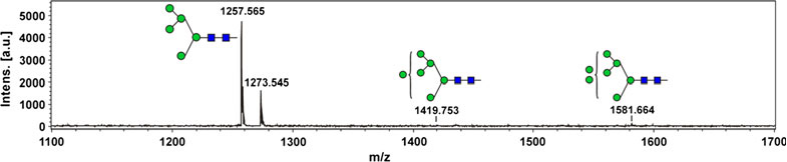
*N*-glycans on *cgl*-IDUA after anti-HRP affinity chromatography identified by MALDI-TOF MS. Glycan cartoons are according to the recommendations of the consortium of functional glycomics (www.functionalglycomics.org).

The purified *cgl*-IDUA sample was subjected to further affinity chromatography with anti-horseradish peroxidase (HRP) antibodies to determine whether the small amounts of recombinant enzyme containing plant complex and hybrid *N*-glycans (approximately 5.5%) could be efficiently removed. As shown in Figure 4 and Table 3, Man5 accounted for 93.8% of the *N*-glycans, while Man6–Man8 accounted for 6.2% of the *N*-glycans. No plant complex or hybrid *N*-glycans were detected. Thus, the anti-HRP chromatography step gave rise to a single dominant *N*-glycan structure of IDUA.

**Table 3:** *N*-glycans identified in *cgl*-IDUA after anti-HRP affinity chromatography

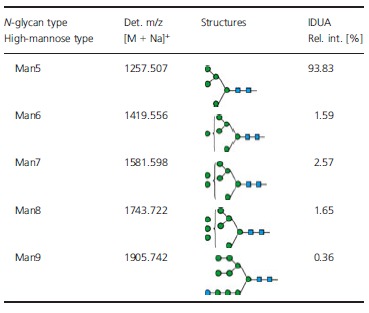

### *Cgl*-IDUA exhibits kinetic parameters that are comparable with the commercial enzyme

**Figure 5:**
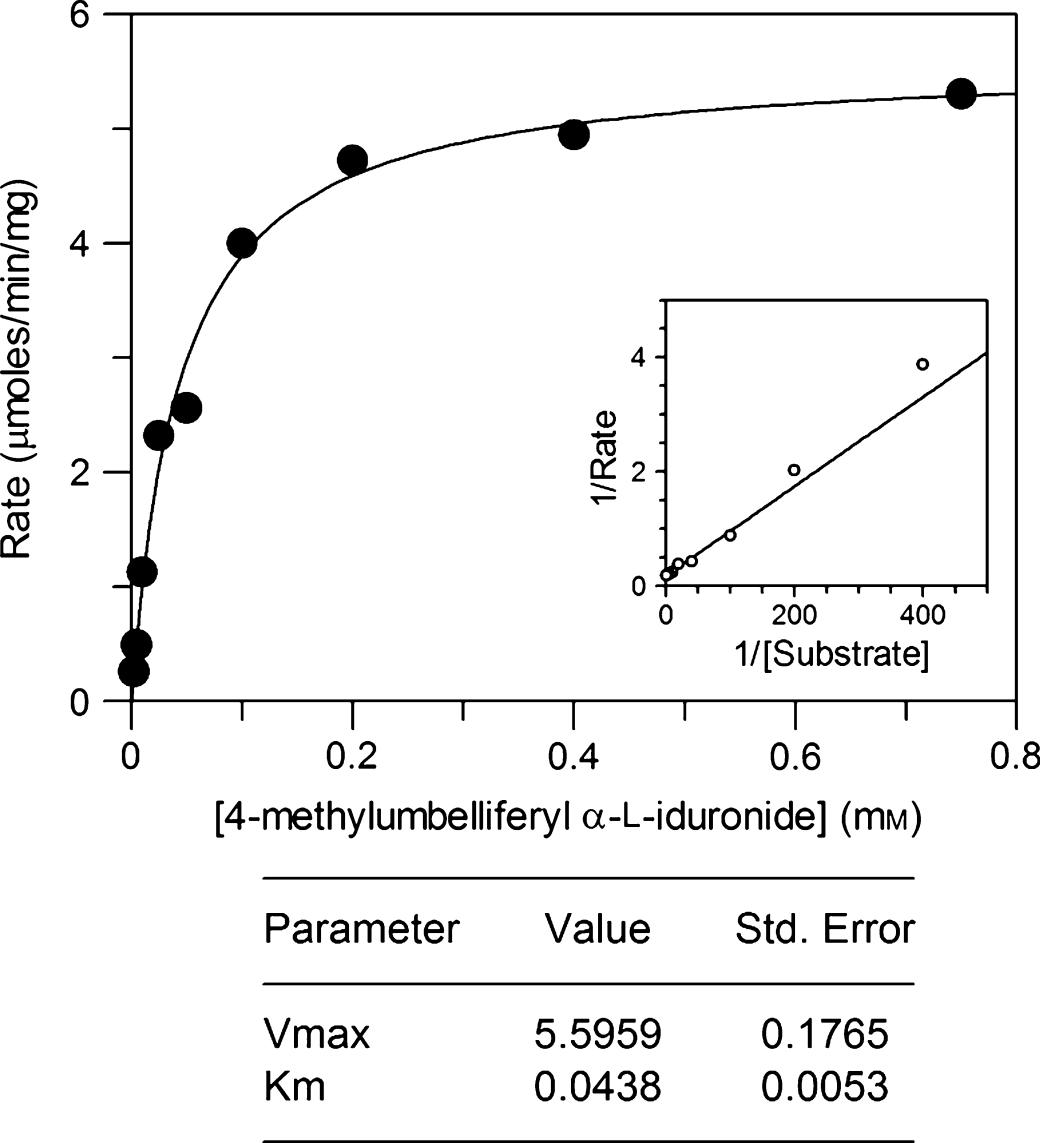
Michaelis–Menten plot for *cgl*-IDUA. Assays were performed in triplicate. The inset on the right is the Lineweaver–Burk plot.

IDUA was purified to homogeneity from T4 seeds (line A4.7) by sequential use of ConA-Sepharose chromatography and anti-IDUA affinity chromatography as described in He *et al*. (2012c) (Figure 1b). Using 4-MUI as substrate, the *cgl*-IDUA exhibited a *K*_m_ of 44 μm and a *V*_max_ of 5.6 μm/min/mg (Figure 5). The values are comparable with those of IDUA produced in CHO cell cultures (Aldurazyme™), for which the *K*_m_ is 24 μm and the *V*_max_ is 3.9 μm/min/mg (He *et al*., 2012c).

### *Cgl*-IDUA is amenable to sequential *in vitro* processing to add the M6P recognition marker

Most lysosomal enzymes including α-L-iduronidase require a M6P tag for efficient uptake and lysosomal delivery in human cells. In mammalian cells, the M6P tag is generated by the sequential action of two enzymes: UDP-GlcNAc:lysosomal enzyme *N*-acetylglucosamine-1-phosphotransferase (also known as GlcNAc-1-phosphotransferase) and *N*-acetylglucosamine 1-phosphodiester α-*N*-acetylglucosaminidase (the uncovering enzyme). The endogenous mammalian GlcNAc-1-phosphotransferase is a heterohexamer comprised of three subunits (α_2_β_2_γ_2_) and is localized within the *cis*-Golgi (Bao *et al*., 1996). GlcNAc-1-phosphotransferase catalyses the transfer of GlcNAc-P from UDP-GlcNAc to C6 of select mannoses of the lysosomal enzyme's high-mannose-type oligosaccharides. The α/β subunits contain the catalytic function of the phosphotransferase and further are responsible for the specificity of the reaction by recognition of a conformation-dependent determinant of the lysosomal enzyme (Kudo *et al*., 2005; Lee *et al*., 2007; Qian *et al*., 2010). The human uncovering enzyme is synthesized as a proenzyme that is activated by the endoprotease furin in the *trans*-Golgi network (Do *et al*., 2002). This enzyme excises the terminal GlcNAc, exposing the M6P recognition marker on lysosomal hydrolases. Both the GlcNAc-1-phosphotransferase (as a α_2_β_2_ heterotetramer) and the uncovering enzyme have been generated as soluble forms (Do *et al*., 2002; Kudo and Canfield, 2006) and so can be used to conduct the *in vitro* phosphorylation of purified recombinant lysosomal enzymes.

**Figure 6:**
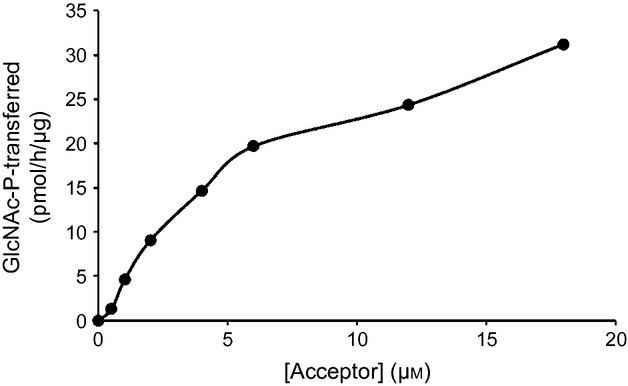
Phosphorylation of *cgl*-IDUA by the GlcNAc-1-phosphotransferase (α_2_β_2_). The activity of the GlcNAc-1-phosphotransferase was expressed as pmoles of [^3^H]GlcNAc-P transferred per h/μg of the GlcNAc-1-phosphotransferase. The graph is based on data from one typical experiment.

We first investigated whether the *N*-glycan terminal mannose residues of the *cgl*-IDUA could be modified *in vitro* using recombinant soluble GlcNAc-1-phosphotransferase (α_2_β_2_). Equal amounts of the phosphotransferase (1.5 μg) were incubated with increasing concentrations of *cgl*-IDUA in the presence of 75 μm UDP-[^3^H]GlcNAc for 1 h, and transfer of [^3^H]GlcNAc-P to *cgl*-IDUA was measured. Figure 6 illustrates the activity of the GlcNAc-1-phosphotransferase towards *cgl*-IDUA based on the data of one representative experiment. Kinetic parameters were determined using the double-reciprocal plot from 3 experiments. *Cgl*-IDUA exhibited a *K*_m_ of 4.3 μm and a *k*_cat_ of 0.10 min^−1^ (*V*_max_ of 21.7 pmol/h/μg).

**Figure 7:**
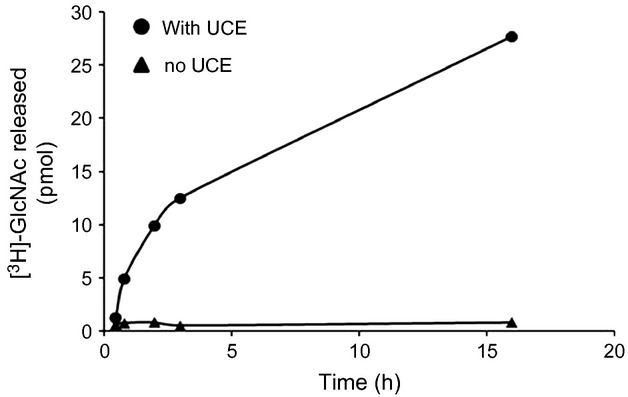
Release of [^3^H] GlcNAc from [^3^H] GlcNAc-P (‘phosphorylated’)-*cgl*-IDUA by the soluble uncovering enzyme. [^3^H] GlcNAc-P-*cgl*-IDUA was incubated with or without 500 ng of soluble recombinant uncovering enzyme. At the times indicated, a 30-μL aliquot was removed, and the amount of free [^3^H] GlcNAc was determined. Each point is the average of two determinations.

Next we examined the efficiency by which the uncovering enzyme cleaved GlcNAc from the phosphorylated *cgl*-IDUA by monitoring the concentrations of free [^3^H]GlcNAc over a time course. The amount of liberated [^3^H]-GlcNAc increased with time (Figure 7), suggesting that the covering GlcNAc of the phosphorylated IDUA can be readily removed by the uncovering enzyme to expose the M6P tag.

## Discussion

In this study, we sought to address the technical challenges of plant-based production of recombinant enzymes as replacement therapeutics for patients with lysosomal storage disease (LSD). To recap, these challenges pertain to the development of economically viable production systems that generate non-immunogenic and functional therapeutics (see Introduction).

### High levels of active α-L-iduronidase (IDUA) are produced in Arabidopsis *cgl* seeds

Arabidopsis seeds have attracted considerable attention as a platform to produce recombinant proteins. In this regard, Arabidopsis has several advantages: its life cycle is short, it can be readily grown in a controlled environment, and compared with several other seed-based systems, it is not a food crop that consequently reduces the regulatory issues surrounding its use as a source of recombinant proteins. In addition, exceptionally high levels of functional recombinant proteins can be accumulated in Arabidopsis seeds. For example, a single-chain variable fragment (scFv) antibody is accumulated to 74 μg/mg of TSP in homozygous Arabidopsis seeds (De Jaeger *et al*., 2002); likewise, the maximal accumulation of antihepatitis A virus antibodies reaches 9.8 μg/mg in dry seeds (Loos *et al*., 2011). In our study, we obtained accumulation levels of IDUA that significantly exceeded our previous reports (Downing *et al*., 2006, 2007), reaching 57 μg/mg TSP (corresponding to 8.0 μg/mg dry seeds) in T4 transgenic Arabidopsis seeds. At this level of expression, one milligram of purified IDUA can be obtained from <1 g of seeds using the chromatographic methods of the present study.

One might be able to apply the lessons learned from Arabidopsis to production of recombinant proteins in a larger seed system, possibly in seeds of a cereal or another dicot. However, efforts to completely knock-out *GnT I* expression in hosts other than Arabidopsis, for example in dicots such as potato and tobacco, lead to a reduction in complex *N*-glycans on endogenous plant proteins (Wenderoth and von Schaewen, 2000), but the extent of silencing would not adequately control the *N*-glycan maturation of a recombinant therapeutic protein. In addition, the yield of IDUA in the stable high-expressing *cgl* Arabidopsis line (A4.7) of the present study is approximately 47 times higher than that of transgenic maize seeds (1.2 μg/mg TSP) (He *et al*., 2012c). The amount of IDUA accumulation in *cgl* seeds also exceeds other seed-based systems; it is about 25–30 times higher than the IDUA levels accumulated by seeds of transgenic tobacco (2.2 μg/mg TSP) and *Brassica napus* (1.9 μg/mg TSP) using the same (arcelin gene) regulatory sequences to drive the human gene expression (Galpin *et al*., 2010).

The development of automated sowing and harvesting of pilled Arabidopsis seeds for bulk production of biomass (Loos *et al*., 2011) represents another advantage for the use of Arabidopsis as a host production system. With the expression level of *cgl*-IDUA at 7–9 μg/mg dry seeds (line A 4.7), the estimated annual production yields of IDUA per square metre (sqm) would range from 1.2 g to 1.6 g (based on 200 mg seeds per plant; 300 plants per sqm; 3 generations per year). A single patient of 40 kg (upper estimate for body weight of 12-year-old patient) (Pastores *et al*., 2007) would annually require approximately 1.2 g of purified IDUA (based on weekly infusions of 0.58 mg/kg of body weight as recommended for Aldurazyme; http://www.aldurazyme.com/global/az_us_home.asp). With an estimated worldwide incidence of MPS I as 1 in 100 000 live births (Muenzer *et al*., 2009) (or potentially approximately 70 000 patients globally), the generation of sufficient *cgl*-IDUA to meet the potential annual market needs would require approximately 140 000 sqm of growth area (approximately 34.6 acres), taking into account the purification yields. Yet this calculation is based on many assumptions, and the true feasibility of using Arabidopsis seeds to produce enzyme therapeutics for patients with LSD will rely upon numerous factors, not the least of which is the associated costs and efficiency of downstream processing (see subsequent Discussion).

### The maturation of the *N*-glycans of recombinant IDUA is avoided in Arabidopsis *cgl* seeds

Aside from achieving sufficient yields of a therapeutic recombinant protein, plant-specific *N*-glycan maturation limits the use of plant-based systems as factories to produce recombinant pharmaceutical proteins; the presence of xylose and fucose residues on ‘matured’ *N*-glycans of the protein may render the product immunogenic if administered parenterally (see Introduction). The human IDUA enzyme has six consensus signals for *N*-linked glycosylation in the ER; these are all utilized in human cells in which the enzyme is targeted to the lysosome via the Golgi complex. In CHO cells hosting production of the recombinant protein, the enzyme is secreted, and again all six sites are used, but the *N*-linked glycans are heterogeneous (Zhao *et al*., 1997) (see Results). To reduce or eliminate plant-specific *N*-glycan maturation, various strategies have been implemented such as a targeted localization of recombinant proteins within the ER or ER-derived compartments, down-regulating glycosyltransferases (e.g. *N*-acetylglucosamine transferase I, α1,3 fucosyltransferases and β1,2 xylosyltransferase) by RNAi technology or by the use of mutants, and expressing human glycosyltransferases (e.g. β1,4-galactosyltransferase) (reviewed in Gomord *et al*., 2010; Kermode, 2012; He *et al*., 2012c). Towards developing various plant-based platforms for the production of recombinant lysosomal enzymes for use in ERT, we have used some of the strategies applied to recombinant human IDUA and/or glucocerebrosidase. Two strategies for ER retention of recombinant IDUA have included KDEL tagging (He *et al*., 2012b) and exploitation of a unique mRNA-targeting mechanism in maize seeds, a process which effectively localizes recombinant IDUA to ER-derived protein bodies (He *et al*., 2012c). Only this latter strategy is effective in controlling the *N*-glycan maturation of IDUA and further has the advantage of yielding a native enzyme with no additional (i.e. foreign) amino acid sequences (He *et al*., 2012c). For example, the KDEL-tagged IDUA of Arabidopsis seeds contains 88% complex/hybrid *N*-glycans (He *et al*., 2012b), while the mRNA-mediated targeting of IDUA to ER-derived protein bodies in maize seeds generates only 8.2% hybrid/complex *N*-glycans (He *et al*., 2012c).

As shown in the present study (and in He *et al*., 2012a for glucocerebrosidase), use of seeds of the Arabidopsis *cgl* mutant is also a viable means to control the *N*-glycan maturation status of recombinant lysosomal enzymes. The *cgl* mutant is defective in the gene encoding the Golgi enzyme *N*-acetylglucosamine transferase I (GnT I) (EC 2.4.1.101) (von Schaewen *et al*., 1993; Strasser *et al*., 2005). GnT I transfers a GlcNAc residue from UDP-GlcNAc to the acceptor substrate Man_5_GlcNAc_2_ to produce GlcNAcMan_5_GlcNAc_2_, which is essential for the subsequent action of α1,3 fucosyltransferases and β1,2 xylosyltransferase to add their respective (plant-specific) sugar residues. The deficiency of GnT I activity in the *cgl* mutant leads to a vast reduction in complex and hybrid *N*-glycans on the plant (endogenous) proteins (von Schaewen *et al*., 1993), although the effectiveness of this strategy for manipulating the *N*-glycan status of recombinant proteins appears to be protein specific (Kermode, 2012) and is considerably more effective for recombinant human IDUA (this study) than for recombinant human glucocerebrosidase (He *et al*., 2012a).

Earlier reports of the *cgl* mutant showed an absence of complex *N*-glycans on endogenous glycoproteins of plant leaf extracts or callus extracts, with the predominant type of *N*-glycan being Man5 (Man_5_GlcNAc_2_) (von Schaewen *et al*., 1993; Strasser *et al*., 2004, 2005). Later, Frank *et al*. (2008) demonstrated the conditional nature of this particular *cgl* mutant (*cgl1* C5), for which a point mutation leading to a destabilized GnT I enzyme accounts for the phenotype (Strasser *et al*., 2005). For example, tunicamycin treatment relieves the ‘folding block’ for the mutant GnT I protein, permitting its transit to the Golgi complex. More recently, our results of the *N*-glycan profiles for *cgl1 C5* mutant-derived recombinant lysosomal enzymes (this study and He *et al*., 2012a,b2012b) suggest that there is a partial restoration of GnT I activity during seed development in this particular mutant. Despite this, we show here that *cgl*-derived IDUA contained predominantly high-mannose-type *N*-glycans (94.5%), with a Man5 structure accounting for 50.6%. The small amount of recombinant IDUA containing complex/hybrid *N*-glycans (5.5%) was efficiently removed by an additional purification step—an affinity chromatography using conjugated antihorseradish peroxidase (HRP) antibodies. Interestingly, this led to a single dominant *N*-glycan of IDUA (Man5: 93.8%) without detectable plant-specific complex/hybrid *N*-glycans (i.e. those containing xylose/fucose residues). Thus, from the point of view of controlling *N*-glycan maturation of IDUA, the strategy of hosting expression in the *cgl1* seed background is very efficient, and the enzyme is stable as a secreted protein.

Incomplete digestion of *cgl*-IDUA by PNGase A allowed the structural analysis of an *N*-glycan at one consensus *N*-glycosylation site (Asn 372); this site possessed a high-mannose-type *N*-glycan (Man 6–9). The high-mannose-type *N*-glycan at Asn 372 is consistently generated on recombinant IDUA regardless of the host production system; it occurs in both Arabidopsis seeds (this study and He *et al*., 2012b for IDUA-KDEL) and in mammalian (CHO) cell cultures (Zhao *et al*., 1997). This suggests that within the folded mature IDUA protein, the *N*-glycan at this site is protected from ‘maturation’ by its inaccessibility to the modifying enzymes of the Golgi complex in both plant and mammalian host cells (i.e. the α1,3 fucosyltransferases and β1,2 xylosyltransferase in the former, and the β1,4 galactosyltransferase and sialyltransferase enzymes in the latter).

### *Cgl*-IDUA can be processed *in vitro* to generate the M6P recognition marker required for lysosomal targeting in human cells

Another challenge associated with plant-based systems for production of LSD enzyme therapeutics is the lack of the endogenous machinery in plant cells required to generate the M6P recognition marker; this tag is ultimately of importance for efficacy of most LSD enzyme therapeutics as their uptake and subsequent lysosomal targeting in human cells occur largely via M6P-receptor-mediated transport systems. The formation of the M6P recognition marker in mammalian cells is catalysed by the sequential action of two enzymes: GlcNAc-1-phosphotransferase and the ‘uncovering’ enzyme. Of the two mammalian/human enzymes involved in creating the M6P tag, the first—the GlcNAc-1-phosphotransferase—is essential for lysosomal trafficking of most lysosomal enzymes as mutations in the gene can cause mucolipidosis II, mucolipidosis IIIA or mucolipidosis IIIC (Kudo *et al*., 2006). No lethal disease has been directly linked with a deficiency of the uncovering enzyme perhaps because the cation-independent M6P receptors can also recognize the disaccharide Man-P-GlcNAc (Olson *et al*., 2010), although a link between mutations in the gene encoding the uncovering enzyme and persistent stuttering has been reported (Lee *et al*., 2011). CHO cell–produced IDUA typically possesses two M6P tags (at Asn336 and Asn451), which are primarily of the biphosphorylated variety: P_2_Man_7_GlcNAc_2_ (Zhao *et al*., 1997).

In the present study, we tested the efficacy of an *in vitro* process to achieve the addition of M6P on *cgl*-IDUA. Kinetic parameters of the soluble GlcNAc-1-phosphotransferase with *cgl*-IDUA as a substrate were determined (*K*_m_ of 4.3 μm and a *k*_cat_ of 0.10 min^−1^). These values are comparable with those of CHO-IDUA treated with alkaline phosphatase (He *et al*., 2012c). The *cgl*-IDUA was amenable to the addition of GlcNAc-P by the soluble recombinant phosphotransferase; through monitoring of the release of [^3^H]-GlcNAc, we further showed that the covering GlcNAc of the phosphorylated IDUA was readily removed by the uncovering enzyme to expose the M6P tag.

Despite this advance in the downstream processing of a plant-made recombinant lysosomal enzyme, the efficiency of the first step of the *in vitro* process (that mediated by the soluble GlcNAc-1-phosphotransferase) clearly needs to be improved. This could be achieved either by modifying the *in vitro* process itself, or, more readily, by modifying the characteristics of the plant recombinant lysosomal enzyme, such as optimizing the number of mannose residues that represent the predominant species of high-mannose-type *N*-glycans. The phosphotransferases of rat liver and simple eukaryotes (*Acanthamoeba castellani*, and *Dictyostelium discoideum*) seem to require *N*-glycans containing Man6 and higher, partly because they contain the required α1,2-linked mannose residues (Couso *et al*., 1986; Ketcham and Kornfeld, 1992). No phosphorylation of *N*-oligosaccharides comprised of Man5 is detected, at least on the target hydrolase that was studied – uteroferrin (Couso *et al*., 1986; Ketcham and Kornfeld, 1992). The *k*_cat_ value of the phosphotransferase for the plant recombinant IDUA as substrate is about 18 times less than that for the mammalian lysosomal protease cathepsin D (Qian *et al*., 2010). The lower *k*_cat_ value may suggest that, as compared to cathepsin D, the *N*-glycans on the *cgl*-IDUA are less efficiently phosphorylated by the soluble phosphotransferase. Yet the *K*_m_ value suggests that the phosphotransferase binds very effectively to the plant IDUA, and part of the efficiency of the phosphotransferase is indeed reliant upon its affinity for the target hydrolase (Qian *et al*., 2010). The extent of phosphorylation of a glycoprotein by the phosphotransferase is influenced by the position of the *N*-glycans relative to the binding site for the phosphotransferase; this modifying enzyme functions best on target lysosomal hydrolases with Man6–Man8 *N*-glycans (Varki and Kornfeld, 1980). This factor may indeed have influenced the extent of GlcNAc-P transfer in the present case as the majority of the *N*-glycans of *cgl*-IDUA are oligomannosidic containing Man5 (Man5 structures account for 50.6%), although approximately 26% are Man 6–8. Perhaps also notable, despite the *N*-glycan at Asn372 of *cgl*-IDUA being of favourable mannose number, this site on IDUA appears to be protected from accessibility to the phosphotransferase, at least *in situ* (Zhao *et al*., 1997).

In summary, we have addressed some of the technical challenges of plant-based production of recombinant enzymes for treatment of lysosomal storage diseases. We are now conducting work to improve the efficiency of the *in vitro* phosphorylation of recombinant IDUA in part through the manipulation of the length of the high-mannose-type *N*-glycans on this human enzyme and by other means.

## Experimental procedures

### Protein extraction and α-L-iduronidase activity assays on transgenic *cgl* lines

Procedures for extraction of total soluble protein from seeds and analyses of α-L-iduronidase (IDUA) activities were as described in the study of Downing *et al*. (2006). Protein amounts were determined using the Bio-Rad DC protein assay kit (Bio-Rad Laboratories, Mississauga, Ontario, Canada) using bovine serum albumin as the standard. The IDUA activities of *cgl* seed extracts were determined at 37 °C and pH 4.5 using 2 mm 4-methylumbelliferyl-α-L-iduronide (4-MUI) as the substrate. Reactions were performed in a total volume of 15 μL, in 0.1 m dimethylglutarate buffer, pH 4.5, 2 mm sodium metabisulfite and 0.7% bovine serum albumin. Reactions were stopped by the addition of 0.1 m glycine buffer, pH 10.7. Fluorescence of the reaction product, 4-methylumbelliferone (4-MU), was determined (λ_ex_ = 365 nm, λ_em_ = 460 nm). The activities were expressed as units/mg TSP, where one unit is defined as 1 nmol 4-MU/min.

### Affinity purification of *cgl*-derived α-L-iduronidase

Affinity purification of *cgl*-IDUA using concanavalin A-Sepharose (ConA-Sepharose) chromatography and affinity chromatography with a monoclonal antibody against IDUA was carried out essentially as described previously (He *et al*., 2012c).

### *N*-glycan profiles of *cgl*-derived IDUA

Purified IDUA (approximately 4 μg) was resolved by 10% SDS-PAGE, and the IDUA protein bands recovered from the gel. *N*-glycans were released from tryptic peptides obtained after in-gel digestion, and graphitized carbon liquid chromatography MS/MS (carbon LC MS/MS) was used for *N*-glycan analysis as outlined in He *et al*. (2012c).

### Anti-horseradish peroxidase affinity chromatography for removal of small amounts of α-L-iduronidase containing plant complex/hybrid *N*-glycans

Approximately 5% of the IDUA derived from *cgl* seeds contained matured (complex or hybrid) *N*-glycans, that is, *N*-glycans with xylose and/or fucose. This small fraction of the IDUA was removed by passing the purified IDUA (a single band on SDS-PAGE) through an anti-horseradish peroxidase affinity column (recycling overnight at 4 °C) according to the method described by He *et al*. (2012c). The column specifically binds to xylose and/or fucose residues (Wilson *et al*., 1998), allowing for the removal of any IDUA containing these sugars from the sample. The resultant IDUA was resolved on 10% SDS-PAGE, and its *N*-glycan composition was determined by MALDI-TOF MS analyses as described in He *et al*. (2012b).

### Determination of kinetics of *cgl*-derived IDUA

The Michaelis–Menten kinetic parameters of 4-MUI cleavage by *cgl*-IDUA were determined using IDUA enzyme purified by ConA-Sepharose and anti-IDUA affinity chromatography. The fluorometric activity assay outlined above was performed using a total volume of 100 μL. The substrate (4-MUI) was used at concentrations of 1 to 800 μm to ensure the coverage of a sufficient range encompassing the *K*_m_. Reactions were started upon the addition of enzyme (0.035 μg per assay) and allowed to proceed for 10 min, over which time it had been determined that the rate of the reaction remained linear. Reactions were stopped by the addition of 0.1 m glycine buffer, pH 10.7. The amount of 4-MU produced at each substrate concentration was determined by measuring the fluorescence and converting the value into a concentration using a standard curve that was prepared using 4-MU concentrations between 0.1 and 125 μm. Rates of catalysis were determined by dividing the reaction time and concentration of the enzyme and fit to a Michaelis–Menten curve using GRAFIT. All measurements were made in triplicate.

### Modification of *cgl*-IDUA by a recombinant soluble α_2_β_2_ GlcNAc-1-phosphotransferase

For *in vitro* phosphorylation of *cgl*-IDUA that had been purified by sequential ConA-Sepharose and anti-IDUA affinity chromatography, the recombinant α_2_β_2_ GlcNAc-1-phosphotransferase (1.5 μg) was added to the reaction mixtures containing various concentrations of *cgl*-derived IDUA in 50 mm Tris–HCl, pH 7.4, 10 mm MgCl_2_, 10 mm MnCl_2_, 75 μm UDP-[^3^H]GlcNAc (1 μCi) and 2 mg/mL bovine serum albumin in a final volume of 50 μL. The assay was carried out as described by Qian *et al*. (2010). Apparent *K*_m_ and *k*_cat_ values were generated from double-reciprocal plots using a least-square approximation for the best fit line. The values were the average of three separate determinations.

### Removal of GlcNAc by the uncovering enzyme

The uncovering enzyme activity assay was performed according to Mullis and Ketcham (1992) and Kornfeld *et al*. (1998). Twenty μmoles of IDUA was phosphorylated by the soluble phosphotransferase overnight at 37 °C under the conditions described above. Phosphorylated *cgl*-IDUA was passed through Sephadex-G-25, and the void volume was collected. The fraction was adjusted to 600 μL with a buffer containing Tris–Maleate pH 6.7, Triton X-100 0.5% and 500 ng uncovering enzyme. The reaction mix was incubated at 37 °C, and at each time point, 30 μL was taken for analysis. After stopping the reaction, the mixture was passed through a 2-mL CM-cellulose chromatography column pre-equilibrated with 50 mm dimethylglutarate (pH 6.0) and eluted with 2 mL of the same buffer. The concentration of [^3^H]-GlcNAc of the eluted fraction was determined by liquid scintillation counting.
